# Supplementary material for: A fault-intrusion-tolerant system and deadline-aware algorithm for scheduling scientific workflow in the cloud
Source: PeerJ Comput Sci. 2021 Nov 2;7:e747. doi: 10.7717/peerj-cs.747 (PMC8576554; doi:10.7717/peerj-cs.747)
Supplement: Supplemental Information 6 [file peerj-cs-07-747-s006.zip › Myproject1.1/docs/org/cloudbus/cloudsim/power/models/class-use/PowerModel.html]

Uses of Interface org.cloudbus.cloudsim.power.models.PowerModel


JavaScript is disabled on your browser.


- Overview
- Package
- Class
- Use
- Tree
- Deprecated
- Index
- Help

- Prev
- Next

- Frames
- No Frames

- All Classes

## Uses of Interface org.cloudbus.cloudsim.power.models.PowerModel

- Packages that use PowerModel

  | Package | Description |
  | --- | --- |
  |  |  |
  | --- | --- |
  | org.cloudbus.cloudsim.examples.power |  |
  | org.cloudbus.cloudsim.power |  |
  | org.cloudbus.cloudsim.power.models |  |
- - ### Uses of PowerModel in org.cloudbus.cloudsim.examples.power

    Fields in org.cloudbus.cloudsim.examples.power declared as PowerModel

    | Modifier and Type | Field and Description |
    | --- | --- |
    |  |  |
    | --- | --- |
    | `static PowerModel[]` | Constants.`HOST_POWER` |
  - ### Uses of PowerModel in org.cloudbus.cloudsim.power

    Methods in org.cloudbus.cloudsim.power that return PowerModel

    | Modifier and Type | Method and Description |
    | --- | --- |
    |  |  |
    | --- | --- |
    | `PowerModel` | PowerHost.`getPowerModel()` Gets the power model. |

    Methods in org.cloudbus.cloudsim.power with parameters of type PowerModel

    | Modifier and Type | Method and Description |
    | --- | --- |
    |  |  |
    | --- | --- |
    | `protected void` | PowerHost.`setPowerModel(PowerModel powerModel)` Sets the power model. |

    Constructors in org.cloudbus.cloudsim.power with parameters of type PowerModel

    Constructor and Description

    `PowerHost(int id,
    RamProvisioner ramProvisioner,
    BwProvisioner bwProvisioner,
    long storage,
    java.util.List<? extends Pe> peList,
    VmScheduler vmScheduler,
    PowerModel powerModel)`

    Instantiates a new host.

    `PowerHostUtilizationHistory(int id,
    RamProvisioner ramProvisioner,
    BwProvisioner bwProvisioner,
    long storage,
    java.util.List<? extends Pe> peList,
    VmScheduler vmScheduler,
    PowerModel powerModel)`

    Instantiates a new power host utilization history.
  - ### Uses of PowerModel in org.cloudbus.cloudsim.power.models

    Classes in org.cloudbus.cloudsim.power.models that implement PowerModel

    | Modifier and Type | Class and Description |
    | --- | --- |
    |  |  |
    | --- | --- |
    | `class` | `PowerModelCubic` The Class PowerModelCubic. |
    | `class` | `PowerModelLinear` The Class PowerModelLinear. |
    | `class` | `PowerModelSpecPower` The abstract class of power models created based on data from SPECpower benchmark: http://www.spec.org/power\_ssj2008/ If you are using any algorithms, policies or workload included in the power package, please cite the following paper: Anton Beloglazov, and Rajkumar Buyya, "Optimal Online Deterministic Algorithms and Adaptive Heuristics for Energy and Performance Efficient Dynamic Consolidation of Virtual Machines in Cloud Data Centers", Concurrency and Computation: Practice and Experience (CCPE), Volume 24, Issue 13, Pages: 1397-1420, John Wiley & Sons, Ltd, New York, USA, 2012 |
    | `class` | `PowerModelSpecPowerHpProLiantMl110G3PentiumD930` The power model of an HP ProLiant ML110 G3 (1 x [Pentium D930 3000 MHz, 2 cores], 4GB). |
    | `class` | `PowerModelSpecPowerHpProLiantMl110G4Xeon3040` The power model of an HP ProLiant ML110 G4 (1 x [Xeon 3040 1860 MHz, 2 cores], 4GB). |
    | `class` | `PowerModelSpecPowerHpProLiantMl110G5Xeon3075` The power model of an HP ProLiant ML110 G5 (1 x [Xeon 3075 2660 MHz, 2 cores], 4GB). |
    | `class` | `PowerModelSpecPowerIbmX3250XeonX3470` The power model of an IBM server x3250 (1 x [Xeon X3470 2933 MHz, 4 cores], 8GB). |
    | `class` | `PowerModelSpecPowerIbmX3250XeonX3480` The power model of an IBM server x3250 (1 x [Xeon X3480 3067 MHz, 4 cores], 8GB). |
    | `class` | `PowerModelSpecPowerIbmX3550XeonX5670` The power model of an IBM server x3550 (2 x [Xeon X5670 2933 MHz, 6 cores], 12GB). |
    | `class` | `PowerModelSpecPowerIbmX3550XeonX5675` The power model of an IBM server x3550 (2 x [Xeon X5675 3067 MHz, 6 cores], 16GB). |
    | `class` | `PowerModelSqrt` The Class PowerModelSqrt. |
    | `class` | `PowerModelSquare` The Class PowerModelSquare. |

- Overview
- Package
- Class
- Use
- Tree
- Deprecated
- Index
- Help

- Prev
- Next

- Frames
- No Frames

- All Classes
